# Ectopic pregnancy in the ampulla of the fallopian tube at 16 gestational weeks: lessons from a case report

**DOI:** 10.4314/ahs.v20i4.47

**Published:** 2020-12

**Authors:** Nnabuike Chibuoke Ngene, Ongombe Lunda

**Affiliations:** 1 Department of Obstetrics and Gynaecology, School of Clinical Medicine, Faculty of Health Sciences, University of the Witwatersrand, Johannesburg, South Africa; 2 Department of Obstetrics and Gynaecology, Klerksdorp Hospital, Klerksdorp, South Africa

**Keywords:** Abdominal pain, ampullary tubal ectopic pregnancy, Bezold-Jarish-like reflex

## Abstract

**Background:**

It is uncommon to find ampullary tubal pregnancy in the second trimester.

**Methods:**

A 35-year-old G4P3 at 16 gestational weeks presented with a day history of sudden severe lower abdominal pain and no vaginal bleeding. The patient had a normal pulse of 82/minutes, haemoglobin concentration of 6.3 g/dl and ultrasonography showed an empty uterus with an alive fetus in the right adnexa. She was provisionally diagnosed to have an abdominal pregnancy.

**Results:**

The patient had an emergency laparotomy where 2.2 L of haemoperitoneum and a slow-leaking right ampullary tubal pregnancy were found. Right total salpingectomy was performed and she had an uncomplicated post-operative follow-up. Histology of the lesion confirmed tubal pregnancy.

**Conclusion:**

The growth of a pregnancy in the ampulla beyond the first trimester is possibly due to increased thickness and or distensibility of the fallopian tube. A tubal pregnancy may present with a normal pulse despite significant haemorrhage.

## Introduction

Approximately 1 – 2% of pregnancies are ectopic in their location.^1^ Of these 90% occur in the fallopian tube^2^ with most (80%) in the ampulla of the tube^2^ and will usually present at approximately 7 gestational weeks.^3^ The index case is a slow-leaking ampullary tubal pregnancy at 16 gestational weeks. Notably, the patient had a normal pulse despite significant haemoperitoneum. The variability in the clinical presentation of ectopic pregnancy may cause delay in recognizing imminent threat to life resulting in increased morbidity and mortality related to the disease particularly in low resource settings where social services and medical infrastructure are inadequate. In this case report, we provide high-quality clinical images of the second trimester ampullary tubal pregnancy, plausible reasons for the pathology and learning lessons.

## Case presentation

**Figure 1:**
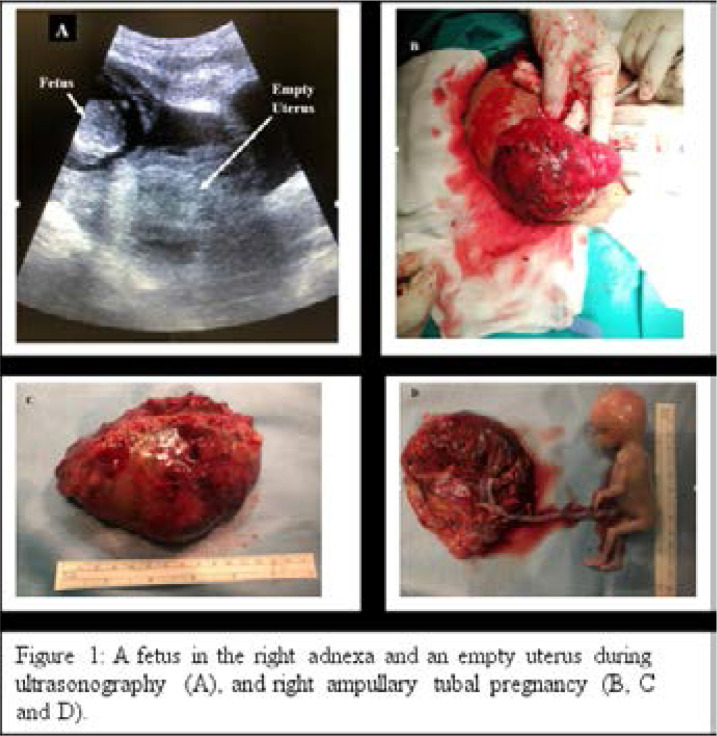
A fetus in the right adnexa and an empty uterus during ultrasonography (A), and right ampullary tubal pregnancy (B, C and D).

A 35-year-old G4P3 lady presented to the emergency room with amenorrhoea of 16 weeks, a day history of sudden severe lower abdominal pain and no vaginal bleeding. Previously, the patient had no symptoms. She is a known cigarette smoker but had no other risk factors for ectopic pregnancy. An evaluation revealed pallor, blood pressure of 90/53 mmHg, a normal pulse of 82/minutes, normal heart, moderate lower abdominal tenderness without guarding, an abdominopelvic mass of 18 weeks uterine size, a haemoglobin concentration of 6.3 g/dl, Rhesus D positive and a positive pregnancy test. Pelvic ultrasonography showed an empty uterus but there was an alive 16-week fetus in the right adnexa (Figure 1). An abdominal pregnancy was suspected, resuscitation was commenced and the patient had an emergency laparotomy where 2.2 L of haemoperitoneum and a slow-leaking right ampullary tubal pregnancy were found (Figure 1). The uterus, ovaries and contralateral fallopian tube were normal. The patient had right total salpingectomy and was transfused 2 units of red blood cell concentrates. She was discharged home two days after the procedure and had a normal post-operative follow-up. Histology of the lesion confirmed a tubal pregnancy with the placenta attached to a distended fallopian tube and a macroscopically normal male fetus. The chorionic villi and the fetal membranes were microscopically normal.

## Discussion

The possible reason for the growth of a pregnancy in the fallopian tube until 16 gestational weeks is the increased thickness and or distensibility of the fallopian tube in the affected patient. This speculation requires further investigation. Unfortunately, there is a limited number of non-interstitial tubal pregnancies beyond the first trimester reported in the literature, and examples include cases managed at 14 weeks, 15 weeks, 16 weeks,^4^ 18 weeks,^5^ 20 weeks,^6^ 42 weeks,^7^ 45 weeks, and 50 weeks^8^ of gestations. Generally, a tubal pregnancy is thought to originate due to an abnormality in the embryo and or a defect in tubal transportation.^1^ In the index case, the fetus appeared macroscopically normal but the tube was distended (Figure 1). The patient smokes cigarette, a life-style which could be the predisposing factor to the ectopic pregnancy.

Some of the lessons demonstrated by this case report are in Box 1 and include that an ampullary tubal pregnancy may remain asymptomatic beyond the first trimester and should be considered as a differential diagnosis of sudden lower abdominal pain and haemodynamic instability developing after the first trimester of pregnancy. Importantly, other conditions such as ruptured liver haemotoma during the second trimester may have a similar presentation as tubal pregnancy.^9^ To aid early diagnosis of ectopic pregnancy, pelvic ultrasonography particularly transvaginal should be offered to all pregnant women early in the first trimester, where possible, to assess if a pregnancy appears normal. The presence of a normal pulse rate instead of tachycardia in a patient with significant haemorrhage is uncommon, noteworthy and may be due to Bezold-Jarish-like reflex which entails a concomitant reduction in pulse and blood pressure because of increased vagal activity following 30% reduction in central blood volume.^10^ An increased parasympathetic response which may be triggered by haemoperitoneum and or emotion may also contribute to the haemodynanic clinical features.

**Box 1:**
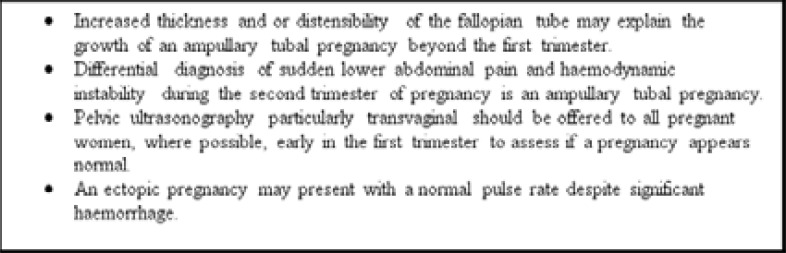
Highlights

## Conclusion

There is variability in the clinical presentation of ectopic pregnancy. Lifestyle modification, commencement of antenatal care early in the first trimester, pelvic ultrasonography particularly transvaginal and prompt treatment are measures that may prevent adverse outcomes from ectopic pregnancies.
